# A214 SIMPLIFICATION OF CARE FOR HCV IS EFFECTIVE DURING THE COVID PANDEMIC: A RETROSPECTIVE STUDY OF HCV TREATMENT UTILIZING THE BRITISH COLUMBIA HEPATITIS C NETWORK

**DOI:** 10.1093/jcag/gwab049.213

**Published:** 2022-02-21

**Authors:** S X Jiang, J Feizi Farivar, J MacIsaac, E Tam, M Choi, P Luyun, H Ko, A Ramji

**Affiliations:** 1 Internal Medicine, The University of British Columbia Faculty of Medicine, Vancouver, BC, Canada; 2 Medicine, University of British Columbia, Vancouver, BC, Canada; 3 Gastrointestinal Research Institute, Vancouver, BC, Canada; 4 Pacific Gastroenterology Associates, Vancouver, BC, Canada

## Abstract

**Background:**

The COVID-19 pandemic has impacted healthcare access, including to curative treatment for hepatitis C (HCV) infection in the form of direct-acting antivirals (DAAs). A 49% decrease in DAA dispensations in Canada during the pandemic has been reported, but little is known about these treated populations.

**Aims:**

To explore the patient characteristics and treatment patterns in those who were treated for HCV during the COVID pandemic.

**Methods:**

A retrospective chart review was conducted at one site of utilizing the British Columbia Hepatitis C Network. Only patients included into the database were analyzed. Patients started on treatment between 03/17/2020-03/16/2021 were included as the “pandemic group” and patients from the 03/17/2019-03/16/2020 were included as a comparison “pre-pandemic group”. Data were extracted for clinicodemographic variables, laboratory investigations, treatment start date, regimen, and sustained virologic response at 12 weeks (SVR12).

**Results:**

97 patients were treated during the pandemic compared to 143 patients the year prior, representing a 32% decline. Patients treated during the pandemic were predominantly new referrals (n=70, 72% vs n=64, 45% pre-pandemic, p<0.01) and had fewer total appointments (median 2 per patient vs 4 per patient pre-pandemic, p<0.01). There was a median of 1 in-person visit and 1 telehealth appointment per patient during the pandemic (vs median 2 per patient of each type pre-pandemic).

Pandemic patients were younger (mean age 56.0 years vs 59.6 pre-pandemic, p=0.04), and a greater proportion were on opioid agonist therapy (28% vs 13% pre-pandemic, p<0.01). Less transient elastography (TE) was performed during the pandemic (69% vs 89% pre-pandemic). Amongst those with TE scores, a lower proportion of those treated during the pandemic were cirrhotic (13% vs 21% pre-pandemic).

During the pandemic, treatment patterns shifted towards more prescriptions for glecaprevir/pibrentasvir (56% of all prescriptions vs 44% pre-pandemic) and sofosbuvir/velpatasvir (37% vs 29% pre-pandemic). There was slightly less use of sofosbuvir/velpatasvir/voxilaprevir at (2% vs 4% pre-pandemic).

The proportion of patients who completed lab work for SVR was similar during the pandemic (n=83/97, 85.6%) compared to pre-pandemic (n=120/143, 83.9%). Similarly, SVR12 remained high during the pandemic at 98.7% (vs 99.3% pre-pandemic). Of all 97 patients prescribed DAAs during the pandemic, 92 (94.8%) completed treatment.

**Conclusions:**

Less persons were treated during the COVID pandemic, which may deter progress towards HCV elimination targets. Very high SVR12 and treatment completion rates during the pandemic suggest that patients can be effectively treated with less pre-treatment investigations and fewer appointments.

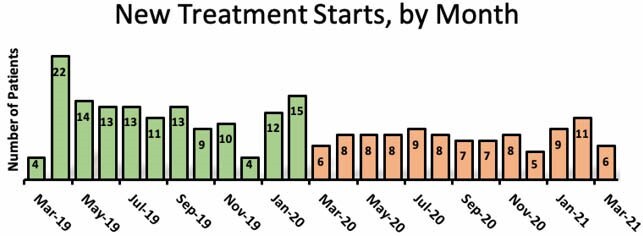

**Funding Agencies:**

None

